# Digital Economy, Environmental Regulation, and Ecological Well-Being Performance: A Provincial Panel Data Analysis from China

**DOI:** 10.3390/ijerph191811801

**Published:** 2022-09-19

**Authors:** Xiaoming Song, Ze Tian, Chenhui Ding, Chao Liu, Wei Wang, Ronggai Zhao, Yingchun Xing

**Affiliations:** 1 Business School, Hohai University, Nanjing 211100, China; 2 Department of Postal Communication Management, Shijiazhuang Posts and Telecommunications Technical College, Shijiazhuang 050021, China; 3 School of Public Administration, Hohai University, Nanjing 210000, China; 4 Foreign Language Teaching and International Exchange Department, Shijiazhuang Posts and Telecommunications Technical College, Shijiazhuang 050021, China

**Keywords:** digital economy, environmental regulation, ecological well-being performance, mediating effects, spatial effects

## Abstract

China is currently in a strategic opportunity period for green and high-quality development, and developing the digital economy is an important choice to achieve environmental pollution control, improve regional ecological efficiency, and enhance social welfare. In this context, the impact of the digital economy on ecological well-being performance and the role of environmental regulation need to be examined. In this study, the super-efficiency SBM-DEA model was used to measure the level of ecological well-being performance in 30 provinces of China from 2011 to 2019. On this basis, the mediating effect model and spatial Durbin model were adopted to explore the transmission mechanism and regional heterogeneity of the impact of the digital economy on ecological well-being performance. The empirical results show that the digital economy significantly contributes to regional ecological well-being performance in China, and there is significant spatial spillover as well. Moreover, the findings still hold under robustness tests. The results also show that environmental regulation is an important transmission path for the digital economy to enhance regional ecological well-being performance, and the impact of environmental regulation on ecological well-being performance varies by region; specifically, the impact in eastern China is positive but not significant. However, the digital economy plays a significant positive role in promoting ecological well-being performance in the central and western regions, and is more obvious in the central region. Finally, suggestions are put forward to enhance the role of the digital economy in regional ecological well-being performance, which is of great significance for promoting green economic growth and high-quality development.

## 1. Introduction

At present, China’s economy has entered a new era of pursuing high-quality internal development, and it is proposed at the national level to firmly establish the socialist concept of an ecological civilization and accelerate the construction of a new pattern of modernization that promotes harmonious development between humans and nature. In the past, China relied on a crude development model with high input and consumption, even at the expense of the environment; failed to consider the finite nature of resources, environmental pollution, and ecological degradation; and tended to neglect social and public utility construction, which was not conducive to improving people’s quality of life and severely restricted sustainable development [1,2,3]. Ecological well-being performance refers to the extent of social welfare improvement at a given level of natural resource consumption and can be measured by the economic, environmental, and health performance of a region. This is in line with the overall requirements of building an ecological civilization in China [4]. In China’s 14th Five-Year Plan, the main binding indicators are related to ecological environmental protection and improvement of residents’ well-being, as a complement to the lack of incentives to protect the environment in China’s long-standing performance appraisal system. The key to the co-integration of economic growth, environmental regulation, and improved ecological welfare lies in technological innovation and the promotion of new business and economic models [5]. At this stage, it is more specifically reflected in the booming digital economy and digital technology in developing countries [6].

Chinese government reports state that the digital economy is a strategic choice for China’s future technological change and creates new opportunities for industrial change, a new round of international competition, and a new round of seizing command of future development. Meanwhile, the breadth and depth of integration between the digital economy and various areas of the real economy continue to expand, which is important in stimulating consumption, boosting investment, and creating jobs [7]. From the international perspective, the Digital Economy Report 2021 released by the UNCTAD, points out that the United States and China are emerging as major leaders in this field, accounting for 90% of the market capitalization of the world’s 70 largest digital platforms, with China’s Tencent and Alibaba among the seven "super platforms". The two countries are leading the way in leveraging data; they have the highest 5G adoption rates, 50% of the world’s hyperscale data centers, 70% of the world’s top AI researchers, and 94% of all funding for AI startups [8]. At the national level, China’s digital economy reached USD 5.4 trillion in 2020, ranking second in the world. The digital economy accounts for 38.6% of China’s GDP and maintains the highest growth rate of 9.7% worldwide, making it a key driver in stabilizing economic growth. In addition, China is globally competitive in digital technologies such as intelligent voice recognition, cloud computing, and some database areas, and Chinese companies rank first in the world with a 32.97% share of 5G patent family declarations [9]. At the regional level, China shows a digital economy scale of over RMB 1 trillion in 13 provinces from 2021, with Beijing and Shanghai leading the country in terms of the share of GDP [10]. As a result, the digital economy has become a new engine for high-quality economic development, the main impetus for deepening supply-side structural reform, and the major driving force in enhancing the resilience of national and regional economic development [11]. It has also become the focus of attention and research by government departments, experts, and scholars.

So, has the digital economy increased the level of ecological well-being performance in China? If this effect is confirmed, what is the mechanism for its operation? What are the differences in the role of the digital economy in ecological well-being performance in terms of its characteristics and spatial laws? Despite the great achievements of the Internet in real life and the gradual emergence of the digital economy as an important component of national economic formation, empirical studies that accurately assess the influence of the digital economy on ecological well-being performance are extremely scarce.

Therefore, this study contributes to the existing literature in three aspects. First, to address the above-mentioned shortcomings, this paper integrates the digital economy, environmental regulation, and ecological well-being performance in the same research framework. Based on a full consideration of their spatial characteristics, this paper enriches and deepens the research content of the digital economy and ecological well-being performance to the maximum extent, providing a new perspective for further research. Second, this paper deeply explores the direct impact of the digital economy on the level of ecological well-being performance and the regional heterogeneity of the spatial effect, and the study of the level of ecological well-being performance is refined using geographical location as a basis for delineation. Third, the indirect impact mechanism of the digital economy on ecological well-being performance is explored based on environmental regulation as a mediating variable at the provincial level in China. The remaining parts of the paper are organized as follows: Section 2 provides a systematic review of the existing literature. Section 3 establishes research hypotheses. Section 4 describes the materials and methods. Section 5 focuses on the empirical tests and results analysis, including benchmark, spatial effects, regional heterogeneity, and robustness tests. Section 6 presents conclusions and policy recommendations.

## 2. Literature Review

The study of ecological well-being performance originated with Daly (1974), who proposed considering both the level of consumption of natural ecological resources and the level of welfare obtained by human beings [12]. Based on Daly’s ideas, Zhu and Zhang (2014) defined ecological well-being performance as “the efficiency of converting ecological resource consumption into social welfare” [13], thus expanding the concept. Ecological well-being performance can be enhanced through either decreasing ecological costs while keeping social well-being, i.e., making a transition toward less material consumption, or prioritizing human development as the final objective of economic activities, i.e., making a transition toward maximizing welfare [14]. The second method achieves greater social benefits through lower ecological cost, thereby achieving a green economy that decouples resource consumption from human welfare.

A series of studies conducted by global scholars on ecological well-being performance focused on three aspects. The first one was the relation between economic growth and ecological well-being performance. Daly (2005) indicated that human society has transitioned from an “empty world” with relatively rich natural capital to a “full world” with scarce natural capital. In a full world, economic growth is not accompanied by increased welfare [15]. The growth of ecological well-being performance has a strong incremental effect on economic growth, and can dominate economic growth, because it takes into account the constraints of the natural environment and the green economic connotation of social well-being with ecological justice-mindedness, based on the traditional efficiency-oriented economic model. Dietz et al. (2012) showed a U-shaped relation between economic growth and ecological well-being performance in 58 countries [16]. Jorgenson (2014) empirically studied the relationship between economic development and human well-being in 106 countries from 1970 to 2010, and they found economic development is conducive to improving human well-being [17]. In addition, the growth of ecological well-being performance from the perspective of sustainable development (Common, 2007; O’Neill, 2015) [18,19], green industrial structure adjustment (Huang, 2014; Jin, 2015) [20,21], and a combination of the two (Raworth, 2012; Daly, 2014; Jorgenson and Dietz, 2015) [22,23,24] has been widely explored. However, many existing studies generally regarded the study countries as spatial entities and clustered their regions together without regard for heterogeneous regional characteristics and spatial spillover, which are prominent attributes of ecological well-being performance growth.

The second aspect of research was the measurement of ecological well-being performance. One measure is the single indicator ratio algorithm, which measures ecological well-being performance by the ratio of human welfare level to ecological resource consumption [25,26], and another is the stochastic frontier model (SFA) and data envelopment analysis (DEA) method based on the evaluation index system of multiple inputs and outputs; for example, Dietz et al. (2012) used the stochastic frontier production function to construct an ecological well-being performance index [16]. Xiao and Zhang (2019) measured the ecological well-being performance of 30 Chinese provinces from 2004 to 2015 using an improved SFA model [27]. Zhang et al. (2018) explored the ecological well-being performance of 82 countries in 2012, and they noted that developed countries and G20 countries had relatively low values [28]. Long (2019) studied the ecological well-being performance of 35 major cities in China based on the super-efficient SBM model, and further analyzed the dynamics of urban ecological well-being performance using the DEA Malmquist Index [29].

The last aspect of research is the influencing factors and spatial effects of ecological well-being performance. Long (2019) found that Chinese tourist cities and economically developed cities were ranked higher in terms of ecological well-being performance, and influencing factors such as urban greenery and compactness were significantly and positively related to the performance level, while the industrial structure and economic contribution were inversely related [29]. Guo and Bu (2018) used a sample of 110 cities in the Yangtze River Basin Economic Zone to analyze the effects of economic scale, industrial structure, level of openness to the outside world, and greening level on ecological well-being performance using the Tobit model [30]. With regard to studies on the spatial effects of ecological welfare performance, Du et al. (2019) and Fang et al. (2019) analyzed regional ecological well-being performance using methods such as coefficient of variation and spatial measures and explored the spatiotemporal divergence pattern, spatial variability, and spatial spillover effects [31,32].

The main shortcomings of the existing studies include three aspects. First, in constructing an evaluation system of ecological well-being performance, the existing studies mainly consider desirable outputs such as economic benefits or social welfare but ignore undesirable outputs that affect real ecological welfare performance such as environmental pollution. Second, most of the studies concentrated on the interaction among environmental regulation, technological innovation, or industrial upgrading and ecological well-being performance, but paid less attention to the digital economy and environmental regulation, and did not positively answer the practical question of whether the digital economy has an effect. Finally, studies on the factors influencing ecological well-being performance were mainly conducted on the basis of regression models, discussing the general impact of independent variables on ecological well-being performance. Nevertheless, spatial effects were not taken into account, therefore possibly resulting in biased regression results, preventing an analysis of the direction and magnitude of the impact of each independent variable on the ecological well-being performance of provinces. Because China’s ecological welfare issue has become increasingly important, each province should make improvements, and sharing experiences among provinces is an effective measure to advance provincial and regional sustainable development. However, few studies have explored the digital economy’s impact on ecological welfare performance. This paper addresses the shortcomings of existing research and improves on them, effectively expanding the research results in this field.

## 3. Theoretical Analysis and Research Hypotheses

In existing research, few authors carried out systematic theoretical analysis and quantitative studies integrating the digital economy, environmental regulation, and ecological well-being performance within the same research framework. Nevertheless, the relationship between these three aspects is becoming increasingly close in China; thus, their internal logic and theoretical basis need to be clarified. In this paper, we propose several hypotheses.

### 3.1. Direct Impact of Digital Economy on Ecological Well-Being Performance

As mentioned above, ecological well-being performance is evaluated by the ratio of a certain natural resource input to the combined social welfare output, and mainly includes economic, environmental, and health performance. Therefore, changes in both resource inputs and welfare outputs affect the level of regional ecological well-being performance. As for expanded outputs, the digital economy’s impact on regional ecological well-being performance is multidimensional and complex. At the micro level, emerging technologies such as the Internet can create an economic environment with economies of scale and scope and long-tail effects at the same time, based on which supply and demand can be better matched and a better price mechanism formed [33]. As a result, the equilibrium of the economy increases, economic welfare output expands, and ultimately the quality of economic growth improves [34]. At the macro level, regions achieve high-quality development through new input factors, new resource allocation efficiency, and new total factor productivity [35,36].

In terms of resource investment, the Chinese government has issued a series of documents on topics such as vigorously developing the digital economy in recent years, and it has further established the role of digital technology as a basic resource and an engine of innovation in its national economic development plan. Digital technology is widely used in transportation, industry, finance, and many other fields, and while it continuously optimizes resource allocation and promotes information sharing, it is also accelerating the demand for technologically innovative data elements. Advances in digital technology have effectively boosted the contrarian growth of digital economy-related industries [37]. The digital economy has become a critical factor in leading the recovery of investment, consumption, and exports, and information consumption has led to the steady recovery of the domestic consumer market. In addition, Chinese provinces and cities are actively promoting digital reform and transformation to provide policy and institutional safeguards and environmental drivers for economic development. As a result, big data and digital technology have become important resources to drive high-quality green development of regional economies and expand social welfare output. On the basis of the above mechanism analysis, we argue that the digital economy can expand the comprehensive output of ecological well-being performance and increase the efficiency of resource input use, thus directly promoting the improvement of ecological well-being performance. Therefore, we propose research Hypothesis 1a.

**Hypothesis** **1a** **(H1a).** *The digital economy plays a direct and positive role in promoting ecological well-being performance*.

To investigate the role of the digital economy in boosting ecological well-being performance, this article further tests the heterogeneity of the digital economy in the eastern, central, and western regions of China. The eastern region has a prominent location advantage; development of its digital economy started earlier and is relatively mature [10]. Relevant digital technology and data resources are widely used in various fields of economy and society. By contrast, the digital economy in the central and western regions started slightly later, but the development speed has been faster and the upside potential is large. Emerging technologies such as big data, the Internet of Things, cloud computing, and artificial intelligence have gradually been applied in several fields, such as industry, education, and healthcare, and they play a huge role in promoting these fields. Meanwhile, education and healthcare are important aspects of regional ecological welfare [38]. Because of the differences in the level and stage of development in the eastern, central, and western regions, as well as different ecological welfare status and government support policies, the digital economy’s impact on regional ecological well-being performance may vary to some extent. Therefore, research Hypothesis 1b is proposed.

**Hypothesis** **1b** **(H1b).** *The driving effect of the digital economy on the level of ecological well-being performance has geographical differences*.

### 3.2. Indirect Impact of Digital Economy on Ecological Well-Being Performance

The improvement of ecological well-being performance is inseparable from the inhibiting effect of environmental regulation on economic development. The effect of environmental regulation on the quality of China’s economic development mainly comes from green development and from improved economic efficiency and social welfare. Environmental regulation has become a key policy to achieve high-quality economic development, and has promoted the upgrading of the regional industrial structure by forcing many highly polluting enterprises to close down [39,40].

Environmental regulation imposes mandatory requirements on specific environmental protection measures such as energy conservation and emission reduction, ecological restoration, and public environmental awareness campaigns through different policy instruments, thus improving local ecological well-being performance by strengthening the effect of policy implementation [6,41]. Moreover, the digital economy relies on innovations in digital technology to broaden the breadth and depth of services and promote operation efficiency. In the process of implementing environmental policies or taking environmental measures, modern digital technologies can be used to effectively resolve information asymmetry, expand service boundaries, increase service supply, and provide the internal impetus for improving ecological well-being performance [39]. As for digital technology innovation and application, environmental information monitoring and disclosure, and industrial optimization and upgrading, regions promote economic development and reduce pollution emissions through the environmental effect of the digital economy, so as to improve their ecological well-being performance. Meanwhile, the level of ecological welfare often determines the intensity of local environmental policy implementation and the process and direction of digital economic development. The digital economy, environmental regulation, and ecological well-being performance have a complex relationship of mutual determination and systematic interaction. Thus, Hypothesis 2 was formulated.

**Hypothesis** **2** **(H2).** *The digital economy can indirectly contribute to regional ecological well-being performance by promoting environmental regulation*.

### 3.3. Spatial Spillover Effects of Digital Economy on Ecological Well-Being Performance

Relying on efficient information transmission, the digital economy compresses the space–time distance, and thus enhances the breadth and depth of regional economic activities. Yilmaz et al. (2002) described the spatial spillover effects of information technology through empirical tests of panel data from 48 states in the United States [42]. Keller (2002) supplemented the discussion of spillover distance from the perspective of knowledge and technology diffusion after Yilmaz et al.’s research [43]. Related studies based on the Chinese context (Bian, 2014; Li and Wang, 2018) [44,45] similarly supported the conclusion that the Internet has spatial spillover. Spatial correlation is one of the prominent characteristics of regional economic activities, and the Internet has spatial spillover effects on regional economic development (Lin et al., 2017; Zhang et al., 2019) [46,47], resource mismatch (Han and Zhang, 2019) [48], and digital finance (Guo et al., 2017) [49]. On the basis of the above analysis, the digital economy’s impact, including the Internet, on ecological well-being performance should also have a spillover effect in space. As a result, we propose research Hypothesis 3.

**Hypothesis** **3** **(H3).** *While promoting the ecological well-being performance in a certain region, the digital economy can also promote the ecological well-being performance of surrounding regions through spatial spillover effects*.

**Figure 1:**
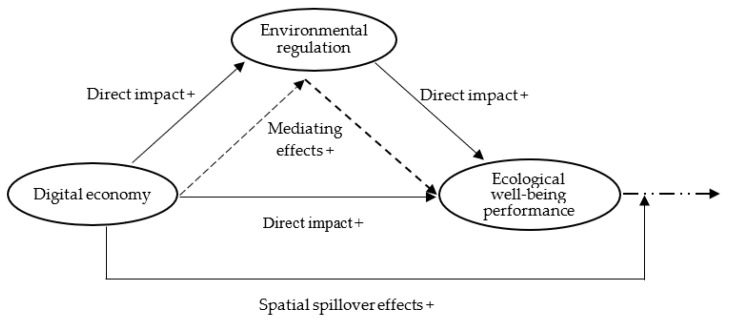
Mechanism of the digital economy acting on ecological well-being performance.

The relationships among the digital economy, environmental regulation, and ecological well-being performance are shown in Figure 1.

## 4. Model Settings and Data Description

This paper’s research objective is to verify the relationship between the digital economy and ecological well-being performance, or, more precisely, to determine whether and how the digital economy affects regional ecological well-being performance. Based on the above theoretical assumptions and analysis, environmental regulation may be an important transmission path for the digital economy to improve ecological well-being performance. Moreover, the improvement of ecological welfare performance has spatial autocorrelation and will be affected by neighboring areas’ development level. The traditional econometric model cannot analyze the spatial effect, and we need to use the spatial econometric model [50]. The spatial Durbin model incorporates the spatial effects of dependent and independent variables at the same time, which can effectively avoid omitting some spatial effects in the spatial error model or the spatial lag model, resulting in errors in the estimation results [51]. Drawing on the studies of Fang et al. (2019) [32] and Wang et al. (2021) [52], this paper analyzes the direct, indirect, and spatial spillover effects of the digital economy on ecological well-being performance and identifies the important role of environmental regulation through the intermediary and spatial models. The research methodology, evaluation index system, and data sources are as follows.

### 4.1. Model Settings

Several basic models are constructed for the direct transmission mechanism to examine the research hypotheses.

(1) $$lnEWP_{it}=\alpha_{0}+\alpha_{1}Dige_{it}+\alpha_{j}Control_{it}+\mu_{i}+\varepsilon_{it}$$

In Equation (1), *lnEWP_it_* represents the ecological well-being performance and *Dige_it_* represents the level of digital economic development of province *i* in period *t*; the vector *Control_it_* describes a group of control variables; *μ_i_* indicates the individual fixed effects of province *i* that do not change with time; and ε*_it_* refers to the random disturbance term.

In addition to the direct effect embodied in Equation (1), for the purpose of exploring the possible indirect mechanism of action of the digital economy on ecological well-being performance, another verification is carried out to confirm whether environmental regulation is an intermediary variable between the two, as described in the previous section. The specific test process is as below. On the assumption that *Dige* passes the significance test of coefficient *α*_1_ of linear regression model (1), linear regression Equation (2) for *Dige* on the intermediary variable of environmental regulation (EG) and linear regression Equation (3) for *Dige* and the intermediary variable *lnEG* on *lnEWP* are established in turn. Among them, the direct effect is represented by the coefficient *γ*_1_, the indirect effect is represented by the product of *β*_1_ and *γ*_2_, and the mediating effect is determined to be either completely or partially mediated one by one in each case. The concrete formulas for the above regression models are as below:

(2) $$lnEG_{it}=\beta_{0}+\beta_{1}Dige_{it}+\beta_{c}Control_{it}+\mu_{i}+\varepsilon_{it}$$

(3) $$lnEWP_{it}=\gamma_{0}+\gamma_{1}Dige_{it}+\gamma_{2}lnEG_{it}+\gamma_{c}Control_{it}+\mu_{i}+\varepsilon_{it}$$

To fully identify the digital economy’s spatial spillover effects on ecological well-being performance, the spatial interaction terms of these two and other control variables are introduced in Equation (1), which is developed into a spatial panel econometric model:

(4) $$lnEWP_{it}=\alpha_{0}+\rho WlnEWP_{it}+\Phi_{1}WDige_{it}+\Phi_{C}WControl_{it}+\alpha_{1}Dige_{it}+\alpha_{c}Control_{it}+\mu_{i}+\varepsilon_{it}$$

where *ρ* denotes the spatial autoregressive coefficient and *W* indicates the spatial weight matrix. The elasticity coefficients of the core explanatory variables and the spatial interaction terms of the control variables are represented by *Φ*_1_ and *Φ*_c_, respectively. Equation (4) is the spatial Doberman model (SDM), which includes the spatial interaction terms of dependent and independent variables. To objectively discuss the digital economy’s impact on regional ecological well-being performance, this paper constructs the adjacent weight matrix, economic-distance spatial weight matrix, and Moran’s index (Moran’s *I*) to test spatial correlation.

The adjacent weight and economic-distance spatial weight matrices are defined as follows:

(1) Adjacent weight matrix

(5) $$W_{ij}=\left\{\begin{array}{ll} 1, & i=j\\ 0, & i\neq j \end{array}\right.$$

This matrix is based on the geographic matrix standard, as shown in Equation (5).

(2) Economic-distance spatial matrix

(6) $$w_{ij}=\left\{\begin{array}{c} 1/\left|{\bar{Y}}_{i}-{\bar{Y}}_{j}\right|\;,i\neq j\\ \;\;\;\;\;\;\;\;\;\;\;\;\;\;\;\;\;\;0\;\;,\;\;\;i=j \end{array}\right.$$

In Equation (6), ${\bar{Y}}_{i}=\frac{1}{T-T_{0}}\sum_{T=t_{0}}^{T}Y_{it}$, ${\bar{Y}}_{i}$ represents the average value of real GDP per capita in region *i*.

(3) Spatial autocorrelation test

In the research process, spatial correlation testing of the observed values between neighboring regions needs to be completed when applying the spatial econometric model. Moran’s *I* and Geary’s *C* are used to examine the spatial correlation of observations. Moran’s *I* can test whether there are analogous, different, or independent relationships between neighboring regions in the spatial system. The formula for the global Moran’s *I* is as follows:

(7) $$I=\frac{n\sum_{i=1}^{n}\sum_{j}^{n}w_{ij}\left(x_{i}-\bar{x}\right)\left(x_{j}-\bar{x}\right)}{\sum_{i=1}^{n}\sum_{j=1}^{n}{\left(x_{i}-\bar{x}\right)}^{2}}=\frac{n\sum_{i=1}^{n}\sum_{j\neq 1}^{n}w_{ij}\left(x_{i}-\bar{x}\right)\left(x_{j}-\bar{x}\right)}{S^{2}\sum_{i=1}^{n}\sum_{j=1}^{n}w_{ij}}$$

In Equation (7), $\sum_{i=1}^{n}{\left(x_{i}-\bar{x}\right)}^{2}/n$, $\bar{x}=\sum_{i=1}^{n}x_{i}/n$; *x_i_* and *x_j_* indicate the observations of regions *i* and *j*, respectively; *n* denotes the number of areas; and *W_ij_* describes the different weight matrix. The value of Moran’s *I* is between −1 and 1, with zero as the cut-off point. A statistical value greater than zero indicates that observations with analogous attribute spatial units are positively spatially correlated. Conversely, a statistical value below zero represents that observations with analogous attribute spatial units are negatively spatially correlated.

### 4.2. Measurement and Description of Variables

#### 4.2.1. Ecological Well-Being Performance Measurement: SBM-DEA Model

Tone first introduced the Super-SBM model in 2002 [53]. He considered slack variables based on a non-radial angle of view and took technical process with the efficiency values. This model has extensive applications in measuring ecological well-being performance [32,54]. Therefore, in this paper, we selected the super-efficiency SBM-DEA model considering undesirable outputs to measure the ecological well-being performance in 30 provinces of China from 2011 to 2019. Ecological well-being performance is the explained variable of this study, and the resource inputs include labor, capital, land, and energy inputs. Among them, labor input is measured by labor stock, capital input by physical capital stock, land input by built-up area, and energy input by a combination of three indicators: energy consumption, total annual water supply, and electricity consumption.

According to the United Nations Development Programme (UNDP), the Human Development Index (HDI) is widely used as the desirable output, includes life expectancy, education, and income dimensions (UNDP, 2016) [55]. Average years of education, average life expectancy, and GDP per capita are used to represent the levels of education, healthcare, and economic development, respectively, in China’s provinces. Undesirable output refers to environmental pollution, mainly including wastewater, exhaust gas, and smoke (powder) dust, which are represented by industrial wastewater, sulfur dioxide, and smoke (powder) dust emissions.

(8) $$Average\;years\;of\;schooling=\frac{P_{Primary\;school\;}\times 6+P_{Junior\;High\;school\;}\times 9+P_{High\;school\;}\times 12+P_{College\;or\;above\;}\times 16}{P_{Primary\;school\;}+P_{Junior\;High\;school\;}+P_{High\;school\;}+P_{College\;or\;above\;}}$$

In Equation (8), *P* represents the number of people in each educational section, with data obtained from the China Education Statistical Yearbook 2011–2019; missing values in the data were linearly interpolated and predicted.

Detailed descriptions of performance evaluation index system of provincial ecological welfare in China are presented in Table 1.

#### 4.2.2. Digital Economy Index Measurement: Principal Component Analysis Method

This paper uses the digital economy as the core independent variable and draws on the idea of Liu et al. (2020) to use Internet development as the basis of measurement [56] and add a digital transaction indicator system. Considering the data availability, we evaluate the comprehensive development index of the digital economy in two dimensions, namely Internet development and digital financial inclusion. The Internet is the carrier and supporter of digital economy development [42]. Referring to Huang’s (2019) approach [57], the Internet development level of each province is measured from four angles: Internet penetration rate, mobile phone penetration rate, employees in related industries, and the outputs of related industries, which are calculated using the number of Internet users per 100 people, the number of mobile phone users per 100 people, the proportion of personnel employed in computer services and software industries to those employed in other urban industries, and the total value of telecommunications business per capita, respectively [58]. Digital financial inclusion is one of the important factors of the digital economy’s development. This paper uses China’s provincial digital inclusive financial index, compiled by Ge et al. (2021), to evaluate the level of digital inclusive finance in various provinces, which is composed of the breadth, depth of use, and degree of digitization [59]. Finally, referring to the study of Zhao et al. (2020), principal component analysis is used to standardize and logarithmically process the above five indicators’ data, leading to the comprehensive digital economy development index, denoted as *Dige* [60].

#### 4.2.3. Mediating Variables

A high-quality development goal demands the establishment of an economic system that enhances green, low-carbon, and circular development, and environmental regulation plays an important role in the whole process. Environmental regulation is the intermediary variable in this study, drawing on the studies of Guo et al. (2018) [61] and Lou et al. (2020) [62]. After taking the logarithm of the ratio of investment in environmental pollution control to GDP, environmental regulation is characterized and denoted as *lnEG*.

#### 4.2.4. Control Variables

Population density: The population density significantly differs among provinces. This is a key factor that influences the output of ecological well-being performance. In this paper we selected the number of people per unit area as the variable for population density, denoted as *lnPDE*.

Level of regional economic development: The economic development level of each province is a fundamental effecting factor in regional ecological well-being performance [52]. In this paper, we use the per capita GDP of 30 provinces to regulate various regions’ economic development disparity, and we characterize the control variable as *lnPgdp*.

Market openness: The proportion of total exports and imports to GDP is used to characterize market openness, and it is logarithmically processed, denoted as *lnOpen*.

Technical level: The ratio of science and technology expenditure to GDP and logarithmic processing are used to characterize the technology level, denoted as *lnTC*.

Degree of greenery: The degree of greenery is characterized by the logarithmic value of green park area per capita, denoted as *lnGree*. Energy consumption is characterized by the ratio of regional coal consumption to total energy consumption and logarithmically processed, denoted as *lnEneg*.

The variables’ detailed descriptions are shown in Table 2.

### 4.3. Data Sources and Descriptive Statistics

#### 4.3.1. Data Sources

To test the impact of the digital economy on ecological well-being performance and analyze the role of environmental regulation, balanced panel data of 30 provinces in China from 2011 to 2019 were utilized in this study. To avoid the influence of missing values, Hong Kong, Macao, Taiwan, and Tibet were excluded. To prevent outliers from biasing the results, the relevant data were logarithmically processed and the tailing process was carried out during data processing. In addition, because of unbalanced regional development, we divided the 30 provinces (cities) into three regions: eastern, central, and western China.

The Dige data were acquired from the annual China Science and Technology Statistical Yearbook, EPS Global Database, and China Provincial Digital Financial Inclusion Index. The lnEWP data and control variable data were derived from the annual China Statistical Yearbook, China Environment Statistical Yearbook, China Energy Statistical Yearbook, and China Provincial Statistical Yearbooks. The few missing values were predicted by the mean value method, interpolation method, and grey prediction model, and the posterior differences of the predicted provinces are all within 0.35, indicating that the prediction effect is good and the model prediction accuracy is high. In addition, for the sake of the credibility and accuracy of regression results, the indicators in the paper which take currency as the unit of measurement, taking 2011 as the base year. Considering that the authentic statistics of municipal units are difficult to get, we chose to use provincial units instead in this article. Making use of 9 years of annual data of the 30 provinces, we acquired a dataset included 270 sample observations, constituting ample data points to verify the above assumptions.

#### 4.3.2. Descriptive Statistics

The descriptive statistics for variables are shown in Table 3. The average value of ecological well-being performance (*lnEWP*) was found to be −1.463, with a standard deviation of 0.886 (maximum value 0.609, minimum value −2.742). This indicates that the level of ecological well-being performance varies widely among different regions, which is consistent with the research findings of Deng et al. (2021) and Wang et al. (2021) [63,64]. The digital economy development index (*Dige*) and environmental regulation (*lnEG*) also show characteristics of small mean values and large standard errors. In terms of control variables, there are also significant differences in the level of regional economic development (*lnPgdp*), market openness (*lnOpen*), and technology (*lnTC*), and the structure of energy consumption (*lnEneg*) in various provinces.

## 5. Empirical Results and Analysis

### 5.1. Benchmark Regression and Mediating Effect Test

#### 5.1.1. Benchmark Regression Results

Table 4 reports the linear estimation results of the impact of the digital economy on regional ecological well-being performance. The fixed-effects model was selected based on the Hausman test. The estimated coefficient of the digital economy index in the benchmark regression and mediating effects (*lnEWP1*) is significantly positive, indicating that the digital economy significantly contributes to ecological well-being performance at the inter-provincial level, thus Hypothesis H1a is verified. There is a positive correlation between provincial population density (*lnPDE*) and ecological well-being performance, but the results are not significant, indicating that ecological well-being performance is not effectively improved with increased population. A possible reason is that with the population growth, people’s resource consumption also rises sharply, while bringing environmental pollution, ecological damage, and other undesirable outputs, to some extent offsetting the growth of ecological welfare, resulting in no significant positive correlation between the two. Regional economic development (*lnPgdp*) passes the significance test at the 10% level, indicating that it will contribute to ecological well-being performance. This is consistent with the expected results in this paper, and also largely in line with the actual provincial situation in China, where more developed regions have better infrastructure, such as education and healthcare, and relatively better well-being. The coefficient of market openness (*lnOpen*) is negative, and passes the significance test at the 10% level, which means that expanding market openness could intensify market competition to a certain extent, and incremental improve the pollution level. The coefficient of technical level (*lnTC*) is negative but not obvious, indicating that increasing technical input does not significantly improve provincial ecological well-being performance. A possible reason is that the transformation efficiency of technical input and the ability of green innovation output need to be further improved. There is a non-significant positive correlation between the degree of greenery (*lnGree*) and ecological well-being performance, indicating that ecological well-being performance is not significantly improved with increased greening degree. There is a negative correlation between energy consumption structure (*lnEneg*) and ecological well-being performance, and it remains prominent at the 1% level. This shows that the extensive use of traditional fossil energy will cause pollution and have adverse effects on residents’ health and life expectancy, thereby reducing the level of regional ecological welfare. This is also the reason why China is vigorously developing its digital economy, promoting industrial restructuring and upgrading, and following the path of green development.

#### 5.1.2. Analysis of Mediating Effect Results

From environmental regulation’s perspective, this paper theoretically analyzes the transmission mechanism of the impact of the digital economy on ecological well-being performance. Next, we selected the intermediary effect model to empirically test the hypothesis of this mechanism, and the regression results are shown in Table 4. The regression coefficients of the environmental regulation and digital economy index in the mediating effects (lnEWP1 and lnEWP2) are positive and significant. Among them, the effect of regression coefficient *β*_1_ of the digital economy on environmental regulation in the mediating effects (lnEG) is positive and significant, indicating that the digital economy could significantly enhance environmental regulation. The wide application of digital technology can improve the efficiency of environmental supervision and governance activities, and it can also force enterprises to control production pollution and increase investment in pollution control. In the lnEWP2 model, both the estimated coefficient *γ*_1_ of the digital economy index (Dige) and the estimated coefficient *γ*_2_ of environmental regulation passed the significance test at the confidence level of 10%, indicating that the enhancement of environmental regulation is the mechanism by which the digital economy promotes ecological well-being performance. Under the premise that the market plays a leading role in resource allocation, the digital economy relies on digital technology innovation to broaden the breadth and depth of services, improve the efficiency of resource allocation, and provide strong support for the government to implement environmental policies or take environmental measures. Reasonable environmental policies can in turn promote the reduction in pollution emissions and improvement of the ecological environment, thereby improving regional ecological welfare performance. This is in line with the findings of Wang et al. (2021) [6] and Xu et al. (2022) [8]. Therefore, Hypothesis H2 is validated.

### 5.2. Analysis of Spatial Spillover Effects

A spatial autocorrelation test of the digital economy development index and ecological well-being performance level is needed before the spatial econometric analysis can be conducted. This paper uses Moran’s I to calculate the spatial effect of each year under the geographical distance matrix, as shown in Table 5. According to Table 5, the values of Moran’s I of the digital economy and ecological well-being performance from 2011 to 2019 were calculated under the adjacent weight matrix (W1) and economic distance matrix (W2). The results show that Moran’s I of the digital economy and ecological well-being performance each year under different matrices reached a significance level of more than 10%. This indicates that the digital economy and ecological well-being performance of China’s provinces have significant spatial clustering characteristics, and there is a positive correlation in spatial dependence. With the promotion of China’s regional coordinated development strategy, there will be orderly flow and spillover between adjacent regions in high-end elements such as digital technology, professional talents, and data resources, thus promoting the development of the digital economy and the improvement of social welfare in the whole region. This is in line with the actual development situation in China’s Beijing–Tianjin–Hebei and Yangtze River Delta regions.

Referring to Elhorst (2014) [65], the SDM model with dual spatiotemporal fixed effects was selected, after the LM test, SDM model fixed effects, Hausman test, and SDM model simplification test. Through the LR test, the spatial Durbin model does not degenerate into SAR and SEM models at the level of 5% at *p* = 0.0157 and *p* = 0.0183, respectively. These results are consistent with the Wald test results, and the spatial Durbin model SDM was selected. Table 6 shows the results of the spatial regression models of the effect of the digital economy on ecological well-being performance under two spatial weight matrices.

In order to compare the robustness of the estimations, we also list the estimation results of the spatial lag model (SAR) and the spatial lag model with dual spatiotemporal fixed effects (SEM). It was observed that the spatial self-regression coefficient of ecological well-being performance in the SDM model was significantly positive, while the spatial interaction coefficient of the digital economy was positive, indicating that the sample not only had an exogenous digital economy interaction effect but also an endogenous interaction ecological well-being performance effect. However, the regression coefficient of spatial interaction terms cannot be directly used to examine the marginal impact of the digital economy on ecological well-being performance, because the analysis of spatial spillover effects between regions by simple point regression will produce false estimates. To avoid such defect, this study used partial differential interpretation of variable changes, that is, the direct and indirect effects were used to explain the influence of independent variables in a certain region on the dependent variables of that region and other regions. As shown in Table 6, both the direct and indirect effects of the digital economy on inter-provincial ecological well-being performance were significant, supporting Hypothesis H3.

### 5.3. Regional Heterogeneity Analysis

The differences in resource endowment, geographical location, and development stage among provinces in China can affect the digital economy index and ecological well-being performance level, which will show significant differences in regional distribution. Based on this, further in-depth discussion is needed to explore the possible heterogeneity of the impact of the digital economy on ecological well-being performance at the provincial level. Therefore, the 30 provinces were divided into eastern, central, and western regions for regression estimation. The results are presented in Table 7. It is observed that the digital economy’s impact on ecological well-being performance in eastern China is positive but not significant. In contrast, the digital economy significantly boosts ecological welfare performance in Midwest China, which is more obvious in the central region. A possible reason for this is that the central region has a higher level of digital economy development, giving it an advantage over the western region, and the digital dividends can be fully realized, so it is more effective in improving ecological well-being performance. Considering the digital economy in the eastern region was developed earlier and the digital dividends have been fully realized, it is rather less effective in promoting ecological well-being performance than in Midwest China with later development, resulting in geographical differences, with the promotion effect being highest in the central region, followed by the western and eastern regions. Therefore, Hypothesis 1b, which posits that the digital economy has a heterogeneous effect on ecological welfare performance, is verified.

### 5.4. Further Expansion: Robustness and Endogeneity Tests

Considering that there may be significant differences in the exogeneity of government policy and its impact on the eastern region, as well as the central and western regions, we used the eastern region as the control group and the central and western regions as the experimental group to study the impact of digital economic development on ecological well-being performance. The G20 Initiative on Digital Economy Development and Cooperation, which was presented at the Group of Twenty (G20) Summit in Hangzhou, China, in 2016, was used as an external policy shock to study the policy effects of digital economy development through the difference-in-difference (DID) method. The specific model is as follows:

(9) $$lnEWP_{it}=\alpha_{0}+\alpha_{1}Treat_{it}*Post_{it}+\alpha_{j}Control_{it}+\mu_{i}+\varepsilon_{it}$$

where *i* represents the province; *t* denotes the time; *lnEWP* refers to the dependent variable, ecological well-being performance; and *Treat* and *Post* represent the regional dummy variable and the time dummy variable, respectively. The eastern region was denoted as 0, and the central and western regions were denoted as 1; they were denoted as 0 before the release of the G20 Digital Economy Development and Cooperation Initiative in 2016 and 1 after. The results are shown in Table 8. It is observed that the DID coefficient is significantly positive, indicating that the digital economy has a significant effect on enhancing ecological well-being performance, which verifies the consistency of the study findings with the hypothesis.

Considering the possible bilateral causality between digital economy development and ecological well-being performance, in this paper we used the selected instrumental variables approach to address the endogeneity problem. Drawing on the method of Huang (2019) et al. [57], we selected the per capita postal service and landline penetration rate as instrumental variables for digital economy development to conduct empirical regressions, which met the requirement of correlation between instrumental and explanatory variables and exclusivity between instrumental and other variables. The F-squared statistic is significantly greater than 10, and judging by the empirical criterion, the instrumental variables are strongly correlated with the endogenous explanatory variables. In the weak identification test for instrumental variables, the Kleibergen–Paaprk F-statistic is greater than the critical value at the 10% level of the Stock–Yogo weak identification test, indicating that the test rejects the original hypothesis and does not have the problem of weak instrumental variables. Therefore, the effect of the digital economy on enhancing ecological well-being performance still holds after accounting for endogeneity (the results are shown in Table 8), and this finding further supports the robustness of the research hypothesis.

## 6. Conclusions

This paper systematically illustrates the mechanism and impact of the digital economy on ecological well-being performance using China’s provincial panel data from 2011 to 2019, based on the direct, indirect, and heterogeneous transmission mechanisms as well as the spatial spillover effect. The results show the following: (1) The digital economy can significantly contribute to improving regional ecological well-being performance. The results remain robust under the robustness and endogeneity tests, indicating that the digital economy has become an important engine to promote environmental pollution control, improve regional ecological efficiency, and enhance social welfare in China. (2) The digital economy can directly contribute to as well as indirectly improve the level of regional ecological well-being performance through environmental regulation, and the direct effect is greater than the indirect effect. (3) There is spatial spillover from the digital economy to ecological well-being performance. The digital economy itself has spatial spillover effects, and while promoting ecological well-being performance in one region, it can also drive the digital economy and ecological well-being performance in the surrounding areas. Using the SDM spatial model, the above results remain robust, but there are differences between the direct and indirect effects under different spatial weight matrices. (4) There is spatial heterogeneity of the effect of the digital economy on ecological well-being performance. Analyzed from the perspective of geographic heterogeneity, the digital economy can promote the level of ecological well-being performance in eastern, central, and western regions of China, and the strength of the driving effects is in the order of central, western, and eastern.

Based on this, the following policy recommendations are proposed to improve the ecological well-being performance of China’s provinces and to coordinate the benign interaction between the digital economy, environmental regulation, and regional ecological well-being performance.

First, dynamic and differentiated digital strategies should be adopted to bridge the regional digital divide. At the present stage, the development level of the digital economy varies greatly among regions in China, resulting in different effects on the improvement of ecological well-being performance. Therefore, the government needs to provide more guidance and comprehensive support and to optimize the allocation of resources related to the digital economy to accelerate its development and growth in the central and western regions and achieve coordinated regional development. Specifically, an active, optimized regional digital economy development policy should be established and a special support fund should be set up as soon as possible. In addition, a comprehensive pilot zone for digital economy development, a digital technology incubation center, and a digital industrial park should be constructed to guide the flow of capital to the critical core aspects of the digital economy and give full play to the allocation efficiency of limited resources. Further, more abundant resources and favorable policies should be directed to the central and western regions to guide the flow of digital elements to these regions with weak digital infrastructure, ultimately promoting coordinated development of the digital economy in all three regions. Further, the eastern region should take full advantage of the role of demonstration and leadership, fully utilize the network effects of the digital economy, break the unbalanced constraints between and within regions, and lay a solid foundation for improving overall ecological well-being performance.

Second, a regional collaboration mechanism for environmental governance should be established to narrow the gap in ecological welfare performance between regions. The central government should strengthen the institutional norms and incentive constraints on local government behavior, and incorporate pollution control, environmental protection, and ecological welfare improvement in the local government performance appraisal system [39]. The government should gradually guide local governments to establish a competition mechanism oriented by green development and ecological welfare performance. Interest orientation is the basis of intergovernmental coordination in regional ecological environment governance. The inter-regional benefit compensation mechanism needs to be established and improved, and should make full use of different regions’ comparative advantages, balance the environmental governance costs of various regions, and strictly regulate the control of benefit compensation funds. This mechanism not only allows the compensation of funds, but also promotes regional industrial upgrading and ecological welfare performance. With digital technology as the link, a collaborative innovation platform covering the five elements of government, industry (enterprises), colleges and universities, research institutions, and intermediaries needs to be established urgently, which will break through the limitations of traditional platforms such as resource innovation, information and knowledge closure, and has the characteristics of openness and sharing. At the same time, a collaborative enforcement mechanism for pollution prevention and control should be established to reduce the negative spatial spillover of environmental regulation, effectively block the transfer of polluting industries from outside the region, and reduce the spillover of pollution to realize improved ecological well-being performance.

Third, suitable environmental regulations should be formulated to create a better policy environment for improving social welfare in various regions. There should be different emphases on environmental regulation in different provinces [66], so that environmental regulation’s effect will be dissimilar in different regions. Specifically, the eastern region should adopt loose environmental regulation policies with incentive effects, utilize digital technology platforms to implement real-time supervision of industries, and give full play to the supervisory role of the public, government, and enterprises. This will be conducive to regional green low-carbon development and high-tech industry upgrading, as well as the improved ecological well-being performance. However, to maximize the push-back effect of environmental regulation, the central and western regions should formulate strict and punitive environmental regulation policies to promote regional industrial upgrading, accelerate the output of industrial green innovation performance, and reduce pollutant emissions. These two regions should combine local natural resource endowments to develop multiple new energy sources, increase the use of clean energy equipment, and gradually improve ecological environment quality. In addition, the government should increase its support for green technology research and development, encourage the application and transformation of enterprises’ innovation achievements and promote the market transaction of technological achievements. Ultimately, this will contribute to improving regional environmental quality and ecological well-being performance.

However, this study also has several deficiencies. First, the measurement of the digital economy index is still being explored, and the construction of an evaluation index system have certain limitations. Second, due to data collection constraints, the sample period spanned from 2011 to 2019. The research results will be more robust with additional data. In response to the current unbalanced and insufficient ecological well-being performance, the digital economy provides an important opportunity for coordinated regional development of ecological well-being performance in China. From the theoretical analysis, the gap in regional ecological well-being performance is bound to be reduced thanks to the inclusive nature of the digital economy, so the question arises as to whether the digital economy can become a driver of regional ecological well-being performance convergence. The convergence mechanism needs to be further studied in the future.

## Figures and Tables

**Table 1:** Performance evaluation index system of provincial ecological welfare in China.

Indicator Category	First Grade Indexes	Second Indexes	Third Grade Indexes	Unit
Input indicators	Resource consumption	Labor input	Labor stock	Million people
		Capital investment	Physical capital stock	Billion
		Land input	Built-up area	Million square kilometers
		Energy inputs	Energy consumption	Million tonnes of standard coal
			Total annual water supply	Million cubic meters
			Annual electricity consumption	Billion kWh
Desirable outputs	Benefit level	Economic benefits	GDP per capita	Yuan
		Social welfare	Average years of schooling	Year
			Average life expectancy	Year
Undesirable outputs	Environmental pollution	Exhaust emissions	Industrial sulfur dioxide emissions	Million tons
		Wastewater discharge	Industrial wastewater discharge	Million tons
		Smoke (powder) dust emissions	Industrial smoke (dust) emissions	Million tons

**Table 2:** Descriptions of variables.

Variable Name	Variable Code	Metrics	Variable Property
Ecological well-being performance	*lnEWP*	Ecological well-being performance index, calculated by SBM-DEA model with undesirable outputs and logarithmic processing	Dependent variable
Digital economy	*Dige*	Digital economy index, calculated by principal component analysis	Independent variable
Environmental regulation	*lnEG*	Amount of investment in environmental pollution control/GDP	Mediating variable
Population density	*lnPDE*	Number of people/provincial area	Control variable
Level of regional economic development	*lnPgdp*	Logarithm of per capita GDP	Control variable
Market openness	*lnOpen*	Total imports and exports/GDP	Control variable
Technical level	*lnTC*	Technology expenditure/GDP	Control variable
Degree of greenery	*lnGree*	Green space per capita	Control variable
Energy consumption	*lnEneg*	Characterized by the ratio of regional coal consumption to total energy consumption and logarithmically processed	Control variable

**Table 3:** Descriptive statistics of variables.

		Obs	Mean	Std	Min	Max
Dependent variables	*lnEWP*	270	−1.463	0.886	−2.742	0.609
Intermediate variables	*lnEG*	270	0.184	0.598	−2.384	1.443
Independent variables	*Dige*	270	0.001	0.984	−0.994	3.493
Control variables	*lnPDE*	270	7.873	0.421	6.858	8.618
	*lnPgdp*	270	1.426	0.511	0.303	2.607
	*lnOpen*	270	1.485	1.304	−0.722	4.673
	*lnTC*	270	−0.992	0.516	−1.893	0.282
	*lnGree*	270	2.538	0.212	1.96	2.984
	*lnEneg*	270	−0.188	0.57	−3.695	0.901

**Table 4:** Estimated impact of digital economy on ecological well-being performance.

Variable	Benchmark Regression	Mediating Effects		
	*lnEWP*	*lnEWP1*	*lnEG*	*lnEWP2*
*Dige*	0.084 ** (2.10)	0.083 ** (2.18)	0.304 * (1.67)	0.075 * (1.97)
*lnEG*				0.027 * (1.95)
*lnPDE*		0.023 (1.62)	0.044 (−0.65)	0.024 * (1.71)
*lnPgdp*		0.036 * (1.75)	−0.021 (−0.21)	0.036 * (1.79)
*lnOpen*		−0.012 * (−1.78)	−0.032 (−0.98)	−0.011 * (−1.66)
*lnTC*		−0.012 (−0.89)	0.007 (0.11)	−0.012 (−0.91)
*lnGree*		0.016 (0.27)	−0.680 ** (−2.39)	0.034 (0.57)
*lnEneg*		−0.146 *** (−4.83)	0.044 (0.31)	−0.147 *** (−4.89)
Constant	−1.463 *** (−255.99)	−1.758 *** (−9.06)	2.353 ** (2.55)	−1.820 *** (−9.31)
N	270	270	270	270
R-squared	0.018	0.139	0.048	0.153

Note: ***, **, and * denote statistical significance at 1, 5, and 10%, respectively, and values in parentheses represent t-statistics or z-statistics.

**Table 5:** Moran’s I of digital economy and ecological well-being performance from 2011 to 2019.

Year	*Dige*		*LnEWP*	
	W1	W2	W1	W2
2011	0.190 **	0.104 *	0.276 ***	0.484 ***
2012	0.181 **	0.105 *	0.257 ***	0.464 ***
2013	0.180 **	0.105 *	0.212 **	0.406 ***
2014	0.176 **	0.110 *	0.194 **	0.378 ***
2015	0.154 *	0.107 *	0.287 ***	0.413 ***
2016	0.150 *	0.106 *	0.229 ***	0.369 ***
2017	0.161 *	0.122 *	0.204 **	0.336 ***
2018	0.171 **	0.157 **	0.212 **	0.340 ***
2019	0.142 *	0.176 **	0.250 ***	0.365 ***

Note: ***, **, and * denote statistical significance at 1, 5, and 10%, respectively.

**Table 6:** Regression results of spatial model of the impact of the digital economy on ecological well-being performance.

Variable	SDM		SAR		SEM	
	W1	W2	W1	W2	W1	W2
*Dige*	0.060	0.098 **	0.091 **	0.094 ***	0.085 **	0.102 ***
	(1.64)	(2.57)	(2.49)	(2.58)	(2.28)	(2.71)
*lnPDE*	0.015	0.014	0.022	0.024 *	0.018	0.023 *
	(1.11)	(0.96)	(1.58)	(1.73)	(1.31)	(1.68)
*lnPgdp*	0.029	0.024	0.037 *	0.038 *	0.036 *	0.038 **
	(1.44)	(1.15)	(1.84)	(1.88)	(1.86)	(1.98)
*lnOpen*	−0.010	−0.007	−0.012 *	−0.013 *	−0.012 *	−0.013 **
	(−1.53)	(−1.11)	(−1.86)	(−1.93)	(−1.82)	(−1.97)
*lnTC*	−0.008	−0.016	−0.013	−0.012	−0.014	−0.012
	(−0.63)	(−1.20)	(−0.95)	(−0.90)	(−1.10)	(−0.89)
*lnGree*	0.132 **	0.153 **	0.009	0.012	0.059	0.052
	(2.02)	(2.27)	(0.16)	(0.21)	(0.88)	(0.79)
*lnEneg*	−0.196 ***	−0.178 ***	−0.150 ***	−0.151 ***	−0.165 ***	−0.162 ***
	(−6.44)	(−5.69)	(−5.10)	(−5.13)	(−5.34)	(−5.31)
rho	0.207**	0.200 **	0.138	0.155 *		
	(2.30)	(2.52)	(1.54)	(1.95)		
W*Dig	0.268 ***	−0.082				
	(2.79)	(−1.28)				
LR_Dire	0.074 **	0.097 **	0.093 **	0.096 **		
	(1.98)	(2.50)	(2.46)	(2.55)		
LR_Indi	0.355 ***	−0.067	0.016	0.018		
	(2.98)	(−0.94)	(1.18)	(1.37)		
LR_Total	0.429 ***	0.030	0.109 **	0.114 **		
	(3.48)	(0.40)	(2.40)	(2.47)		
N	270	270	270	270		
R-squared	0.168	0.234	0.168	0.221		

Note: ***, **, and * denote statistical significance at 1, 5, and 10%, respectively, and values in parentheses represent t-statistics or z-statistics.

**Table 7:** Regional heterogeneity test for impact of digital economy on ecological well-being performance.

Variable	Eastern Region	Central Region	Western Region
*Dige*	0.099 (1.60)	0.686 *** (4.22)	0.137 ** (2.46)
*lnPDE*	0.036 (1.31)	0.016 (0.18)	−0.023 (−1.09)
*lnPgdp*	0.034 (0.83)	−0.097 (−0.91)	0.047 (1.57)
*lnOpen*	−0.010 (−0.72)	0.024 (0.50)	−0.015 * (−1.68)
*lnTC*	−0.011 (−0.35)	−0.071 (−0.83)	−0.001 (−0.07)
*lnGree*	−0.534 *** (−3.31)	1.258 *** (4.47)	−0.152 ** (−2.39)
*lnEneg*	−0.251 *** (−5.73)	0.244 * (1.89)	−0.065 (−0.88)
Constant	−0.435 (−0.89)	−4.715 *** (−4.93)	−0.762 * (−1.91)
Observations	99	72	99
Number of obs	11	8	11

Note: ***, **, and * denote statistical significance at 1, 5, and 10%, respectively, and values in parentheses represent t-statistics or z-statistics.

**Table 8:** Robustness tests for impact of digital economy on ecological well-being performance.

Variable	Substitution Variable	DID	2SLS
*Dige*	0.108 *** (2.85)		0.124 * (1.74)
DID		0.070 *** (3.27)	
*lnPDE*	0.022 (1.51)	0.016 (0.92)	0.028 (0.23)
*lnPgdp*	0.036 * (1.73)	0.023 (0.84)	−0.229 (−1.63)
*lnOpen*	−0.011 (−1.61)	−0.006 (−0.82)	0.162 *** (3.17)
*lnTC*	−0.015 (−1.08)	−0.009 (−0.75)	−0.106 (−0.92)
*lnGree*	0.054 (0.81)	0.093 (0.63)	−0.509 ** (−2.17)
*lnEneg*	−0.156 *** (−5.09)	−0.206 *** (−5.94)	−0.433 *** (−3.78)
*lnSoc*	−0.104 (−1.60)		
Constant	−1.618 *** (−6.49)	−1.886 *** (−3.89)	−0.490 (−0.42)
N	270	270	270
R-squared		0.246	0.211

Note: ***, **, and * denote statistical significance at 1, 5, and 10%, respectively, and values in parentheses represent t-statistics or z-statistics.

## Data Availability

The data presented in this study are available from the corresponding author.
